# Imatinib alters cell viability but not growth factors levels in TM4 Sertoli cells

**Published:** 2016-09

**Authors:** Seyyed Mohammad Reza Hashemnia, Somayeh Atari-Hajipirloo, Shiva Roshan-Milani, Nasim Valizadeh, Sonya Mahabadi, Fatemeh Kheradmand

**Affiliations:** 1 *Department of Biochemistry, Tehran University of Medical Sciences and Urmia University of Medical Sciences, Urmia and Tehran, Iran.*; 2 *Department of Physiology, Cellular and Molecular Research Center, Urmia University of Medical Sciences, Urmia, Iran.*; 3 *Hematology-Oncology and Stem Cell Transplantation Research Center, Tehran University of Medical Sciences, Tehran, Iran.*; 4 *Department of Biochemistry, Cellular and Molecular and Solid Tumor research centers, Urmia University of Medical Sciences, Urmia, Iran* *.*

**Keywords:** *Cell viability*, *Imatinib mesylate*, *Sertoli cells*, *Platelet derived growth factor*, *Stem cell factor*

## Abstract

**Background:**

The anticancer agent imatinib (IM) is a small molecular analog of ATP that inhibits tyrosine kinase activity of platelet derived growth factors (PDGFs) and stem cell factor (SCF) receptor in cancer cells. However these factors have a key role in regulating growth and development of normal Sertoli, Leydig and germ cells.

**Objective:**

The aim of this study was to determine cell viability, PDGF and SCF levels in mouse normal Sertoli cells exposed to IM.

**Materials and Methods:**

In this experimental study, the mouse TM4 Sertoli cells were treated with 0, 2.5, 5, 10 and 20 μM IM for 2, 4 or 6 days. The cell viability and growth factors levels were assessed by MTT and ELISA methods, respectively. For statistical analysis, One-Way ANOVA was performed.

**Results:**

IM showed significant decrease in Sertoli cell viability compared to control group (p=0.001). However, IM increased PDGF and SCF level insignificantly (p>0.05).

**Conclusion:**

Results suggested that IM treatment induced a dose dependent reduction of cell viability in Sertoli cells. It seems that treatment with this anticancer drug is involved in the fertility process. Further studies are needed to evaluate the role of PDGF and SCF in this cell.

## Introduction

Imatinib mesylate inhibits growth factor signal transduction mediated by tyrosine kinases like abl (the Abelson proto-oncogene), platelet-derived growth factor receptor (PDGF-R), stem cell factor (SCF) receptor (c-kit) and breakpoint cluster region (bcr). This drug is used in the treatment of chronic myelogenous leukemia, gastrointestinal stromal tumors and a number of other malignancies and hypereosinophilic syndrome (1, 2).

As growth factor signal transduction pathways that are up-regulated in many types of tumor cells, perform critical functions in normal cells, imatinib (IM) may affect normal cells like Sertoli cells. Platelet-derived growth factor (PDGF) is a growth-regulatory molecule that stimulates chemotaxis, proliferation and metabolism primarily of mesenchymal origin cells. PDGF and PDGF-R have been reported to be expressed in the human testis and possibly could affect testis developmental processes, spermatogenesis and male infertility. It plays a critical role in the pathogenesis of serious diseases, such as cancer (3). A Sertoli cell is part of a seminiferous tubule that helps in spermatogenesis process and sperm production. Its main function is to maintain the developing of sperm cells through the spermatogenesis stages (4). Sertoli cells are required for male sexual development and can synthesize several secretory products such as PDGF (5, 6). In addition, it was shown that PDGF motivates a key enzyme of estrogen biosynthesis (aromatase), mainly localized in Sertoli cells suggesting an additional site of regulation of Sertoli cell by PDGF (3, 7).

SCF and c-KIT signaling also play a main role in cell fate decisions, specifically controlling cell proliferation, differentiation, survival, and apoptosis (8). SCF is secreted by Sertoli cells and found on their cell membrane (9-11). As cell proliferation is crucial for the production of male germ cells and fertility, decreased expression of SCF or c-KIT in the testis and aberrant SCF/c-KIT signaling are associated with male infertility (12, 13). 

Gambacorti-Passerini *et al* showed that IM was associated with a reduction in testicular hormones (14). Besides, several studies have been reported that IM reduces testosterone production in the testis (15-17). However, Schultheis *et al* showed that IM administration (150 mg/kg/day) for 2 months had no effect on spermatogenesis in a leukaemic mouse model (18).

To our best knowledge there are rare studies about the effects of IM on Sertoli cells. The aim of this study was to investigate the effect of IM on expression of these factors in TM4 Sertoli cultured cells.

## Materials and methods

**Drug**

1 mM stock solution of IM (provided by Mr Aziz mohammadi, Farayand-shimi Co) was prepared in distilled sterile water and stored at -20^o^C. In this study IM was used at concentrations 2.5, 5, 10 and 20 μM for 2, 4 or 6 days. Controls were prepared by treating cells with culture medium containing the equal volume of distilled water. Each condition was present in triplicate. 

**Cells**

In this experimental study, the mouse normal testis TM4 Sertoli cell (genetic Laboratory of Tehran University) was cultured in 90% DMEM-F12 medium (PAA, UK), 5% fetal bovine serum (PAA, UK), 5% horse serum (PAA, UK) and 100 unit/ml penicillin. Cells were incubated at 37^o^C in a humidified 5% CO_2_-enriched atmosphere. The controls were treated with a DMSO vehicle at a concentration equal to that of the drug-treated cells.

**Cell viability assay**

Viability of cells in different groups was determined by using MTT proliferation assay kit (Cayman chemical, USA). TM4 cells (5000 per well) were treated with mentioned concentration of IM in 100 μl of cultured media. 

At the end of specified periods, 10 μl of MTT reagent was added per well and the plates were incubated at 37^o^C for 3 hrs. MTT-containing media were then removed and the reduced formazan dye was solubilized by adding 100 μl of Crystal Dissolving Solution to each well. The absorbance was measured at 570 nm using an ELISA microplate reader (StatFax 2100, Awareness Technology Inc.). Cell survival rate was reported as percentage and calculated as follows: (OD values of the experimental samples/OD values of the control) ×100.

**Growth factors determination**

Concentration of PDGF and SCF in different groups were assayed using Human/Mouse PDGF-AA Immunoassay and Mouse SCF Immunoassay kits (R&D Systems, Inc. Minneapolis, MN, USA) respectively. Each sample was replicated three times.

**Statistical analysis**

All data were presented as mean±SD for at least three separate experiments for each treatment. Statistical significance of differences between mean values was analyzed by one way ANOVA followed by Tukey’s HSD post-hoc test using SPSS software (Statistical Package for the Social Sciences, version 16.0, SPSS Inc, Chicago, Illinois, USA). The level of significant difference was set at p<0.05.

## Results

**The effect of IM on cell viability**

**Figure 1:**
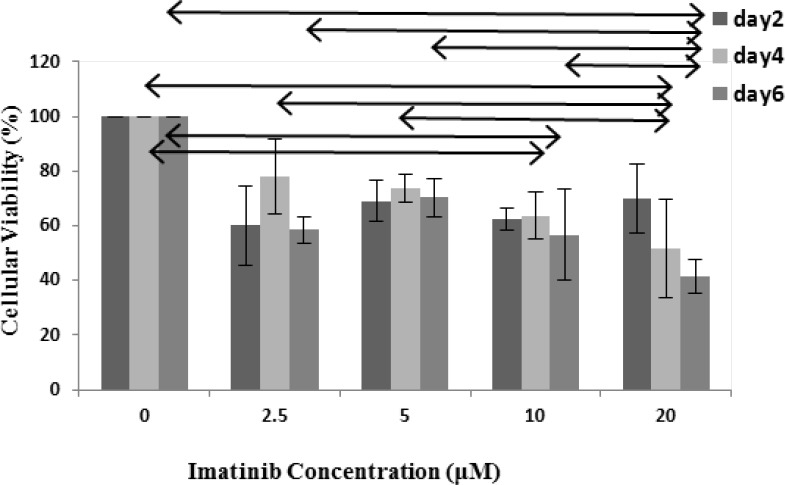
Effect of different concentrations of IM on Sertoli cell viability in vitro

Treatment of IM produced dose dependent growth inhibition in Sertoli cells (Figure 1). The cell viability decreased on days 4 and 6. IM decreased the cell viability of Sertoli cells in higher dose, namely 20 µM of IM resulted in 59% reduction after 6 days compared with the controls (p<0.001), and 10 and 5 µM of IM caused 43% and 30% reduction in cell viability (p<0.001, p=0.01), respectively. MTT data demonstrated that the long term, and high dose IM treatment was significantly more effective on inhibiting Sertoli cell growth compared to untreated group. 

**The effect of IM treatment on PDGF and SCF levels**

Increasing drug concentration in cultured media and also duration of treatment had no statistically significant effect on PDGF (p>0.05). However in all treated doses PDGF level increased by increasing duration of treatments from 4-6 days. SCF level increased in treated cells by increasing duration of treatment especially at concentrations higher than 2.5 μM, but it was not statistically significant (p>0.05).

**Figure 2:**
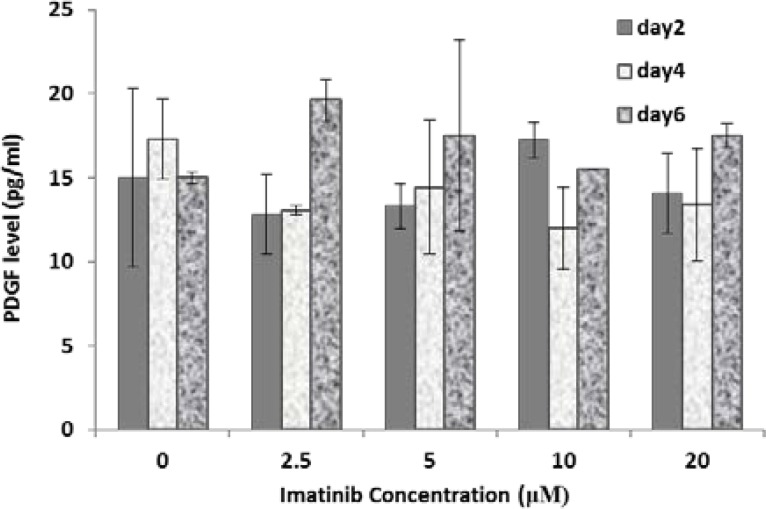
PDGF level in Sertoli cells treated with different concentrations of IM for different periods (2, 4 and 6 days). Data represent mean values ± SD of three analyses. Mean values were compared using ANOVA. The P value was compared to untreated group, (p<0.05

**Figure 3:**
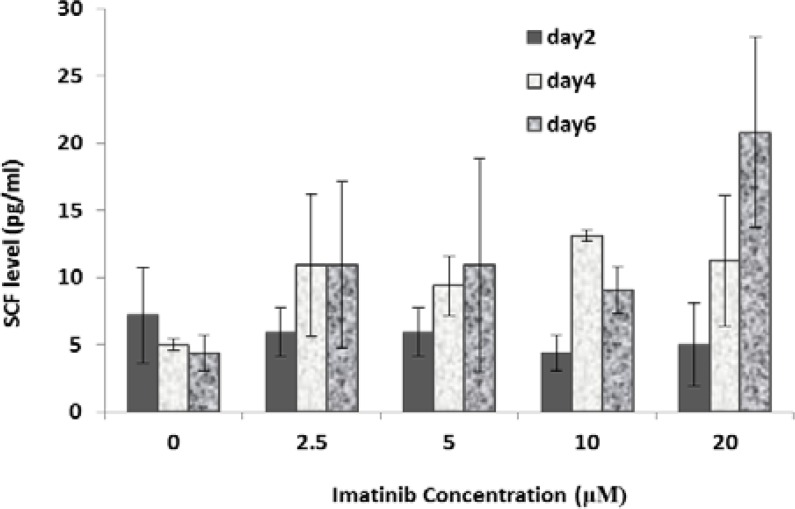
SCF level in Sertoli cells treated with different concentrations of IM for different periods (2, 4 and 6 days). Data represent mean values ± SD of three analyses. Mean values were compared using ANOVA. p<0.05 according to Tukey’s post hoc test

## Discussion

Anti-cancer drugs which used in chemotherapy are one of the major causes of infertility. The effects of anticancer drugs are based on their anti-mitotic action such as spermatogenic cells (19-21). In this study the effect of IM as an anti-cancer drug on Sertoli cell viability was investigated. IM treatment caused a dose dependent inhibitory reduction in Sertoli cells. Comparing these results with our previous study, it appears that IM is less efficient in decreasing cell viability in Sertoli cell than in Leydig cell (22). 

However, according to Nurmio *et al* study Sertoli cells do not express c-kit or PDGFR receptors, therefore these cells continued to proliferate normally after IM treatment in rats (23). Basciani *et al* also reported no obvious defects in Sertoli cells in IM treated rats (24). In contrast, our data showed that 20 μM IM decreased Sertoli cell viability in vitro significantly on days 4 and 6. Of course our study was an in vitro study and reduction in cell viability has been seen in long term high dose (10 and 20 μM) IM treatment. As final number of Sertoli cells in the adult testis correlates with total sperm output, the decrease of cell viability might result in fertility problems (25). 

It has been shown that IM treatment for three days during the first postnatal week decreased the proliferation of PDGF-stimulated human testicular peritubular cells and also decreased type A spermatogonia but increased germ cell apoptosis (23, 26). Prasad *et al* reported that IM does affect sperm morphology significantly, but this effect is reversible once the drug is withdrawn (17). Yaghmaei *et al *suggested dose and time dependent effect of IM on spermatogenesis (27). Additionally, it has been shown that treatment of cancer with IM may have suppressed effects on testicular development by inhibiting SCF/c-kit and PDGF signaling pathways (2). 

To the best of our knowledge, there has been no study about the effect of IM on PDGF and SCF levels in normal Sertoli cells. In our study PDGF and especially SCF levels increased slightly by increasing duration of treatment, however it was statistically insignificant. Previously, we have shown that IM increases PDGF level significantly in Leydig cell (22). We have also previously reported increasing of PDGF receptor phosphorylation in Leydig cell (28). 

In this study we have shown that the amount of increase in SCF level was more than PDGF level in Sertoli cells. However according to our former results PDGF level was increased in Leydig cells but SCF level did not change (22). PDGF is produced by Sertoli cell, however these cells are the main source of SCF. As C-kit is expressed on differentiating spermatogonia and Leydig cells, then IM effect on Sertoli cells can affect spermatogonia and Leydig cells by altering SCF level (29). 

Yan *et al* reported that SCF/c-kit interaction functions as a survival signal for mature Leydig cells, play an important role in the gonocyte migration process, Leydig cell differentiation, subsequent testosterone production, and spermatogenesis (30-32). However SCF alteration in our study was very slight and may not have an important effect on spermatogonia and Leydig cells.

Taken together, it seems that Sertoli cells are less vulnerable than Leydig cells especially at lower doses. However as these cells are supportive cells of the seminiferous tubules, IM can affect spermatogenesis by mediating effect of Sertoli cells. Besides, direct effect of IM on Leydig also should be considered in evaluating the effect of IM on fertility potential (22).

## Conclusion

In conclusion, the present in vitro study on Sertoli cells demonstrated that IM significantly reduces the cell viability of these cells. Our study also presents that PDGF and SCF might have slight role in anti-proliferative effects of IM. Since testicular cells are affected by anticancer drugs, therefore comprehensive studies seem to be necessary for evaluating the effect of IM on spermatogesis and infertility. 
